# Editorial: Bacteriophages, a weapon against animal bacterial pathogens and biofilms

**DOI:** 10.3389/fvets.2025.1625678

**Published:** 2025-05-30

**Authors:** Yan Zhou, Hang Yang, Shaohui Wang

**Affiliations:** ^1^Jiangsu Key Laboratory for Food Quality and Safety-State Key Laboratory Cultivation Base of Ministry of Science and Technology, Institute of Food Safety and Nutrition, Jiangsu Academy of Agricultural Sciences, Nanjing, China; ^2^State Key Laboratory of Virology and Biosafety, Wuhan Institute of Virology, Chinese Academy of Sciences, Wuhan, China; ^3^Shanghai Veterinary Research Institute, Chinese Academy of Agricultural Sciences, Shanghai, China

**Keywords:** bacteriophages, phage-derived proteins, infection, biofilms, bacterial pathogens

## Introduction

In the field of bacteriology, the rapid rise of drug-resistant bacterial infections and the lack of effective antimicrobial agents have necessitated the development of alternative therapies. Bacteriophages, or phages, are bacterial viruses that are abundant and diverse, with lytic phages possessing the ability to invade bacterial cells, disrupt their metabolism, and cause cell lysis. These properties make lytic phages potential biocontrol agents for treating bacterial infections in humans and animals, particularly against drug-resistant bacteria. Phages have, therefore, global attention. Phage-derived proteins, such as endolysins, holins, polysaccharide depolymerases, and peptidoglycan hydrolases, have also demonstrated antibacterial activity. However, despite the increasing interest in phage-based biocontrol strategies, there is a need for more comprehensive studies to better understand the interplay between phages or phage-derived proteins and bacteria or biofilms. Thus, in this Research Topic, four original research articles as well as one review article were published.

## Organization of the Research Topic

This Research Topic aims to publish original research and review articles that explore the application of phages or phage-derived proteins as antimicrobial agents against animal bacterial infections or biofilms. Given the escalating global challenge of drug-resistant bacteria, they highlight the growing importance and potential of phages and phage-derived proteins in human health, aquaculture, and agriculture. Five articles focused on phages and phage-derived proteins research across various pathogenic bacteria species, including *Aeromonas hydrophila, Proteus mirabilis, Salmonella abortus equi, Staphylococcus aureus*, and *Streptococcus bovis/Streptococcus equinus* complex (SBSEC).

*Aeromonas hydrophila*, a Gram-negative facultative anaerobic bacterium, is a common pathogen of freshwater farmed animals. It is pathogenic to a wide range of fish, amphibians, and reptiles and can cause systemic and ulcerative infections, including septicemia, gill rot, and kidney disease. Wang et al. characterized a novel virulent *Aeromonas hydrophila* phage phiA051 isolated from aquaculture water. They found the genome of phage phiA051 has high similarity to many prophages of *Aeromonas* spp., suggesting its prophage origin. *Proteus mirabilis* is a Gram-negative, rod-shaped bacterium widely found in natural environments. It is known for causing a range of severe illnesses in mammals, particularly urinary tract infections (UTIs). Wu et al. evaluated the therapeutic efficacy of phage P2-71 against *Proteus mirabilis in vivo* and *in vitro* environments. Results revealed that *in vivo*, phage treatment significantly lowered bacterial concentrations in the urine on Days 1 and 3 (*p* < 0.0001), achieving a maximum reduction of 4.602 log_10_ CFU/mL. Their findings demonstrated that phage P2-71 is a promising alternative therapy for UTIs caused by MDR *Proteus mirabilis*. *Salmonella abortus equi* is a prominent pathogen known to cause abortion in equidae (horses, donkeys, and mules). Phage depolymerase breaks the bacterial polysaccharide structure, releasing repeating polymer units and facilitating phage infection of bacterial cells. Zhao et al. investigated a *Salmonella abortus equi* phage 4FS1 and its depolymerase, Dpo36. Their findings confirmed that Dpo36 effectively disrupts biofilms and exhibits potent antimicrobial activity against *S. abortus equi* in both *in vitro* and *in vivo* settings. *Staphylococcus aureus* is one of the most important zoonotic pathogens and can be transmitted to humans through the meat diet routes, causing necrotizing pneumonia. Phage lysin is a cell wall hydrolytic enzyme synthesized by the phage gene coding in the later stage of phage infection of bacteria. Lysin can target peptidoglycan in bacterial cell walls, causing peptidoglycan lysis and resulting in bacterial cell wall rupture, leading to bacterial death. Zhang et al. investigated the therapeutic efficacy of phage lysin LysGH15 against necrotizing pneumonia caused by *Staphylococcus aureus* in a rabbit model. The study revealed that LysGH15 treatment effectively reduced the number of bacteria in infected rabbit lungs, inhibited the production of bacterial toxins, reduced the production of cytokines, significantly improved the pathological manifestations of lung tissues, and ultimately increased the survival rate. Their results suggest that LysGH15 has the potential to be used as a novel antimicrobial agent for the treatment of necrotizing pneumonia caused by *Staphylococcus aureus*. *Streptococcus bovis/Streptococcus equinus* complex (SBSEC) comprises eight (sub)species, with several opportunistic pathogenic members. These SBSEC species are associated with metabolic disorders in ruminants, resulting in economic losses to the global livestock industry. Moreover, the emergence of antimicrobial resistance (AMR) in SBSEC strains, particularly against commonly used antibiotics, poses serious concerns to the livestock industry. Park et al. reviewed SBSEC and their phages in ruminants. In the review, authors discussed the taxonomy, AMR characteristics, and diversity of SBSEC, and the potential of SBSEC-specific phages, focusing on recent advances in basic research and biotechnological applications in ruminants. They pointed out the potential and limitations of phage therapy and highlighted that developing phage cocktails, screening for strictly lytic phages, and exploring strategies to minimize resistance development, such as combination therapies with antibiotics or phage-derived enzymes, could enhance the efficacy of phage therapy.

## Conclusion

In conclusion, these articles underscore the significance and essential role of phage and phage-derived protein-based biocontrol strategies as novel and effective therapeutic agents in eliminating and reducing pathogenic bacteria.

